# New insights into the role of cellular senescence and rheumatic diseases

**DOI:** 10.3389/fimmu.2025.1557402

**Published:** 2025-05-16

**Authors:** Jianting Wen, Jian Liu, Lei Wan, Fanfan Wang

**Affiliations:** ^1^ Department of Rheumatology and Immunology, First Affiliated Hospital of Anhui University of Chinese Medicine, Hefei, Anhui, China; ^2^ Institute of Rheumatology, Anhui Academy of Chinese Medicine, Hefei, Anhui, China; ^3^ Anhui Province Key Laboratory of Modern Chinese Medicine, Department of Internal Medicine Application Foundation Research and Development, Hefei, Anhui, China

**Keywords:** cellular senescence, rheumatic diseases, chronic inflammation, treatment strategy, TCM

## Abstract

Rheumatic disease is a chronic inflammatory disease that imposes significant societal and economic burdens. Accumulating evidence has demonstrated that cellular senescence plays an important role in inflammation-induced rheumatic diseases. Due to the lack of effective therapies, there is an urgent need for a deeper understanding of the etiopathogenesis of rheumatic diseases. In this review, we systematically summarized the role of cellular senescence in rheumatic diseases. We first focused on the mechanisms and hallmarks of cellular senescence, and then summarized evidence that can induce or aggravate cellular senescence, as well as related signaling pathways. Next, we discussed the mechanisms of interaction between cellular senescence and rheumatic diseases. Additionally, we focused on and elucidated the mechanisms and impacts of chondrocyte senescence and mesenchymal stem cell (MSC) senescence in osteoarthritis (OA) and systemic lupus erythematosus (SLE), respectively. Finally, we highlighted the potential of therapies targeting senescent cells in rheumatic diseases as a strategy, especially the multi-target effect of traditional Chinese medicine.

## 1 Introduction

Rheumatic diseases are a class of chronic, multi-system autoimmune diseases with a complex and multifactorial etiology, including rheumatoid arthritis (RA), osteoarthritis (OA), ankylosing spondylitis (AS), systemic lupus erythematosus (SLE), systemic sclerosis (SS), etc. (1, 2). Besides, dysfunctional joints and skin, and the extra-articular manifestations include cardiac, ocular, lung, cutaneous, gastrointestinal, neurological, and renal involvement in rheumatic diseases (3, 4). Furthermore, long-term oral treatment can exacerbate liver and kidney function damage and significantly affect patient’s quality of life (5, 6). The clinical treatment of rheumatic diseases is limited because of its complex pathogenesis.

Senescence is a cellular state marked by irreversible cell cycle arrest, accompanied by distinct secretory phenotypes and metabolic reprogramming, which critically contribute to aging and chronic disease progression. Chronic inflammation is a core feature of the pathogenesis of rheumatic diseases (7, 8). Cellular senescence is a process accompanied by low-grade chronic inflammation (9). Senescent cells secrete pro-inflammatory mediators, which in turn affect cellular senescence (10, 11). Conversely, inflammation plays a pivotal role in driving cellular senescence. This phenomenon occurs when inflammation is excessively regulated and is known as inflammageing (12–14). Growing studies have shown that cellular senescence affects cell function through the secretion of senescence-associated secretory phenotypes (SASP), which exacerbate inflammatory responses and tissue damage; additionally, cellular senescence’s significant role in rheumatic disease pathogenesis is also elucidated (15).

Recent advancements in the study of cellular senescence and rheumatic diseases underscore the necessity of a comprehensive review in this area. In this review, we explored the history, mechanism, and hallmarks of cellular senescence, as well as its role in the pathology of rheumatic diseases; additionally, we summarized evidence that can induce or aggravate cellular senescence, and related signaling pathways. By clarifying the connection between cellular senescence and rheumatic diseases, this review aimed to provide valuable insights into the treatment of rheumatic diseases, as targeting this process holds promise for improving the outcomes of rheumatic disease patients.

## 2 Cellular senescence

### 2.1 Hallmarks of cellular senescence

**Figure 1:**
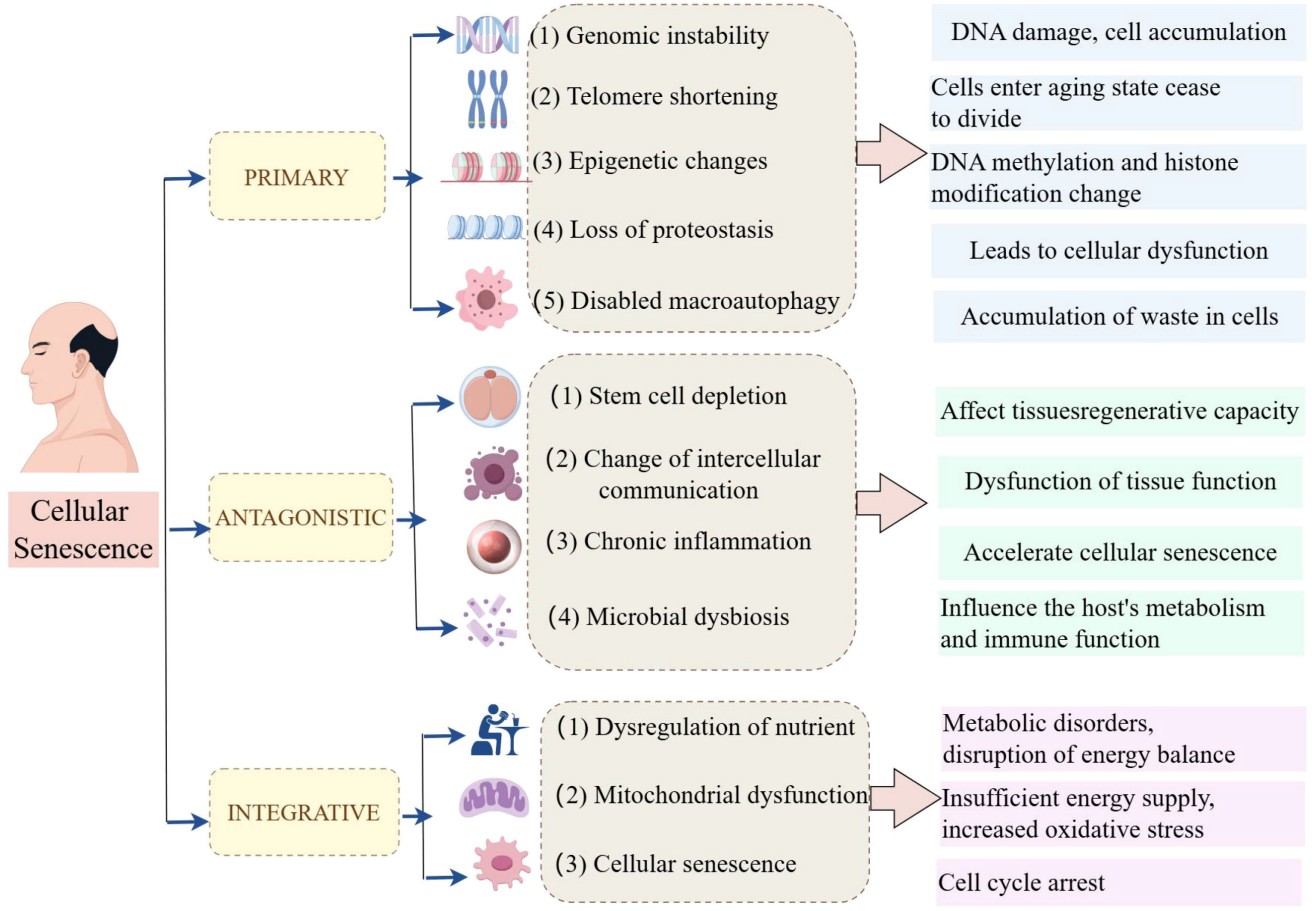
The hallmarks of cellular senescence by Figdraw.

In 1961, Leonard Hayflick and Paul Moorhead first described the phenomenon known as “cellular senescence”, a form of “senescence at the cellular level”; additionally, they stated that primary human fibroblasts have a restricted lifespan in culture, with approximately 50 cell divisions (16). Cellular senescence is a complex biological process involving multiple cellular and molecular mechanisms (17, 18). In recent years, researchers have summarized twelve major characteristics of cellular senescence, which not only help us understand the biological basis of senescence but also provide new perspectives for developing anti-senescence therapies (**Figure 1**).

The primary characteristics of hallmarks of cellular senescence are shown as follows. (1) Genomic instability is an important characteristic of cellular senescence. As cells age, DNA damage gradually accumulates within the cell, leading to genomic instability. This instability may result in the loss of cellular function and even contribute to the development of diseases such as cancer. (2) Telomere shortening (TS) is another significant characteristic of cellular senescence. Telomeres are protective structures at the ends of chromosomes whose length gradually decreases during cell division. When telomeres become too short, cells enter a senescence state and cease to divide. (3) Epigenetic change is also an important sign of cell senescence. As age increases, epigenetic markers (such as DNA methylation and histone modification) change, affecting gene expression and cellular function. (4) The loss of protein homeostasis, characterized by an increase in protein misfolding and aggregation, is also a hallmark of cellular senescence. This phenomenon leads to cellular dysfunction and is associated with various age-related diseases. (5) Dysfunction of macroautophagy also plays an important role in cell senescence. Macroautophagy is a process in which cells clear damaged or excess organelles, and its dysfunction may lead to the accumulation of waste in cells.

The main antagonistic hallmarks were shown as follows. (1) Stem cell depletion is an important manifestation of cellular senescence. As age increases, the number and function of stem cells gradually decrease, affecting the regenerative capacity of tissues. (2) The change in intercellular communication is also a characteristic of cell senescence. Changes in intercellular signaling may lead to the dysfunction of tissue function. (3) Chronic inflammation, also known as inflammation senescence, is an important characteristic of cell senescence. Chronic inflammation state accelerates cell senescence and is associated with various age-related diseases. (4) Microbial dysbiosis is also considered a hallmark of cellular senescence. Changes in the gut microbiome may affect the host’s metabolism and immune function, thus accelerating the senescence process.

Furthermore, the integrative factors mainly included 3 items. (1) Dysregulation of nutrient sensing is a feature of cellular senescence. Decreased responsiveness to nutritional signals may lead to metabolic disorders and disruption of energy balance. (2) Mitochondrial dysfunction plays a key role in cellular senescence. Mitochondria are the energy factories of cells, and their dysfunction leads to insufficient energy supply and increased oxidative stress. (3) After being subjected to various stresses, cells will enter an irreversible state of growth arrest, known as cellular senescence. These features provide a framework for understanding cellular senescence and developing potential targets for new anti-senescence strategies.

Senescence is a cellular state characterized by growth arrest, anti-apoptosis, SASP, and DNA damage response (DDR) (19–21). These characteristics are expressed and mechanized differently in different biological contexts. First, cells enter an irreversible cell cycle arrest, typically accompanied by activation of DDR. DDR is the mechanism by which cells recognize and repair DNA damage; if the damage cannot be repaired, the cells will enter a senescent state. Additionally, the sustained activation of DDR is associated with TS, and telomere damage leads to cellular senescence while remaining active during the senescence process (22). Second, compared to apoptotic cells, senescent cells indeed exhibit significant anti-apoptotic characteristics. This anti-apoptotic property is mainly due to the significant increase of anti-apoptotic proteins (such as BCL-2) in senescent cells. The increase in this protein leads to cell resistance to apoptosis, thereby promoting the accumulation of senescent cells. Additionally, senescent cells avoid cell death by modulating the expression of apoptosis-related genes, thereby accumulating in the body. Senescent cells still maintain metabolic activity and exhibit a characteristic known as SASP even after experiencing cell cycle arrest. SASP leads to the secretion of various pro-inflammatory cytokines, including IL-1, IL-6, IL-8, proteases, and matrix metalloproteinases (23). SASP production is closely related to the continuous activation of DDR and plays an important regulatory role in the senescence process. Finally, the role of DDR in cellular senescence is not limited to DNA repair but also involves the regulation of the cell cycle and signaling (24). Abnormal activation of DDR can lead to genomic instability, which is critical in senescence and cancer development. These characteristics interact with each other through complex molecular mechanisms, which jointly affect the fate of cells and the health of organisms.

Biological markers that reflect direct evidence of cellular senescence have not yet been identified, but several markers have been used to indirectly detect senescent cells (25). The most widely used marker of senescent cells is senescence-associated beta-galactosidase (SA-beta-Gal), a lysosomal hydrolase whose activity is significantly increased in senescent cells (26). The activity of SA-β-Gal is typically detected under conditions of pH 6.0, making it one of the classic methods for identifying senescent cells. However, the expression of SA-β-Gal is not always directly related to cellular senescence. For example, some studies have found that SA-β-Gal activity also appears in certain healthy non-proliferative cells. Therefore, although is an important senescence marker, it should be used in conjunction with other indicators to more accurately assess cellular senescence status (27). Histone γ-H2AX (a replacement histone), the second most common marker of cellular senescence after SA-β-Gal, is phosphorylated at the C-terminal serine-139 of the H2AX histone (28). Histone γ-H2AX is the most sensitive marker of double-stranded DNA breaks (DSBs) and TS. The number of γ-H2AX foci increases in damaged and senescent cells in most tissues and species both *in vivo* and *in vitro* (29). As an important marker in the early cellular response to DNA DSBs, γ-H2AX’s role in cellular senescence has attracted significant attention. Studies have shown that γH2AX formation is associated with cell cycle arrest and apoptosis, which may be a protective mechanism adopted by cells when confronted with irreversible DNA damage. Additionally, γH2AX may also participate in regulating senescence-related signaling pathways, thereby affecting cell fate (30).

Senescence-associated heterochromatic foci (SAHF) are densely packed nuclear structures, which contribute to the maintenance of cellular senescence by silencing proliferation-related genes (31, 32). Their formation involves key chromatin remodeling factors, notably the retinoblastoma (Rb) protein and BRG1, whose interaction is essential for SAHF assembly (33). Moreover, the p16INK4a gene, also known as CDKN2a, encodes a cyclin-dependent kinase inhibitor that induces cell cycle arrest, contributing to senescent cell accumulation (34, 35). Similarly, p21, a downstream effector of p53, inhibits CDK activity during G1/S and G2/M transitions (36, 37). Sustained expression of p21 promotes senescence as a protective response to chronic stress, with its expression level and localization influencing its regulatory function (38). These pathways are highly relevant to the pathogenesis of rheumatic diseases and may offer potential targets for therapy.

### 2.2 Stressors and cellular senescence

#### 2.2.1 Inflammation induces cellular senescence

**Figure 2:**
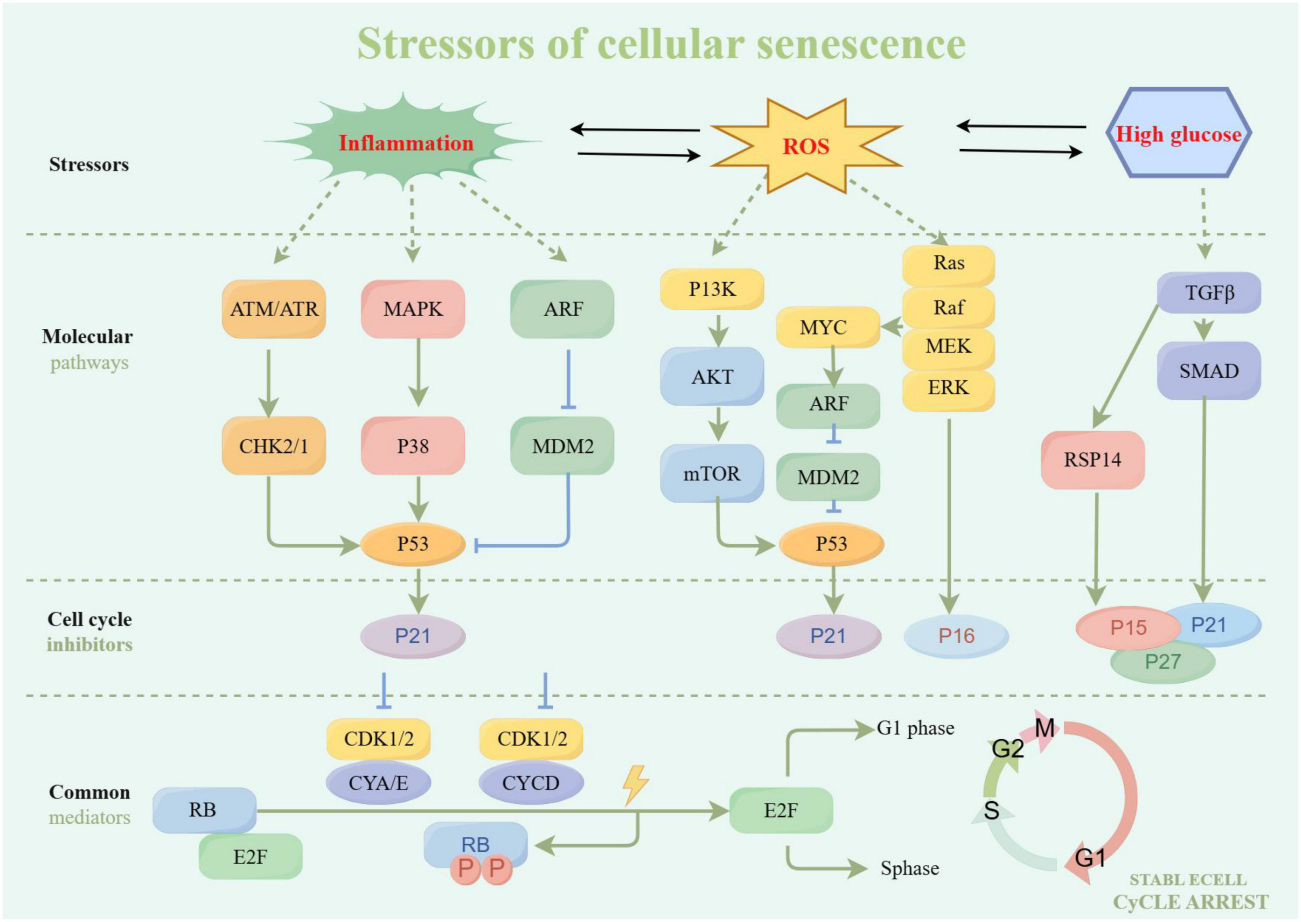
Stressors and cellular senescence by Figdraw.

Inflammation is a critical factor that can induce cellular senescence, which is a state of stable cell cycle arrest typically accompanied by a pro-inflammatory phenotype (39, 40) (**Figure 2**). Inflammatory signals can trigger senescence, and senescent cells can in turn promote inflammation through SASP. This creates a feedback loop that can lead to chronic inflammation, commonly referred to as “inflammaging”, which is a hallmark of senescence (41). For instance, the cGAS-STING pathway is activated by cytoplasmic chromatin fragments in senescent cells, leading to the production of type I interferons and other inflammatory mediators, thereby linking DNA damage responses to inflammation and senescence (42). Moreover, senescent cell accumulation in tissues maintains a pro-inflammatory environment, contributing to cellular senescence-related pathologies. Clearing these cells has been shown to alleviate inflammation and restore tissue homeostasis, highlighting the potential therapeutic benefits of targeting senescent cells to mitigate inflammation-induced tissue damage (43).

#### 2.2.2 Reactive oxygen species induces cellular senescence

The ROS generated during oxidative stress is a potent trigger for cellular senescence whereby it irreversibly limits cell proliferation and these senescent cells secrete various SASP, further inducing the senescence of adjacent cells (44). The accumulation of ROS can lead to oxidative damage to cellular components (such as DNA, proteins, and lipids), thereby triggering the senescence program (45). This process is often associated with the activation of specific signaling pathways and the expression of senescence-associated markers. Specifically, one of the key pathways involved in ROS-induced cellular senescence is the p53/p21 signaling. The presence of ROS can lead to the activation of p53, a tumor suppressor protein that regulates the expression of cyclin-dependent kinase inhibitor p21 (46). Upregulation of p21 results in cell cycle arrest. Additionally, ROS can activate other signaling pathways, such as the IL-6/STAT3 pathway, which is implicated in the induction of cellular senescence in nerve cells (47). ROS accumulation promotes the activation of this pathway, contributing to senescence. Furthermore, ROS is involved in the regulation of autophagy and lysosomal function, which are critical processes in the maintenance of cellular homeostasis. In the context of bleomycin-induced cellular senescence, ROS has been shown to mediate lysosomal membrane permeabilization and inhibit autophagy, leading to the accumulation of damaged cellular components and the promotion of senescence (48). These findings highlight the complex interplay between ROS, cellular senescence, and various cellular pathways.

#### 2.2.3 High glucose induces cellular senescence

Both extracellular and intracellular metabolic disturbances significantly promote cellular senescence. For example, extracellular high glucose refers to an increase in glucose levels in the extracellular environment, which can induce cellular senescence through oxidative stress and inflammatory pathways. Conversely, intracellular high glucose refers to intracellular metabolic dysregulation, which independently contributes to senescence via mechanisms such as hexosamine pathway activation or advanced glycation end-products. Numerous studies have demonstrated that high glucose stimulation induces early cellular senescence (49, 50). For example, it has been shown that high glucose microenvironment markedly decreases miR-30a-5p expression in HMEC-1, elevates SA-β-gal and p21 levels, fosters cellular senescence, and hinders cell proliferation, migration, and vasculogenesis (51). Moreover, studies have shown that high glucose can induce cellular senescence through multiple signaling pathways. For example, high glucose promotes the senescence of mesenchymal stem cells (MSCs) via the Akt/mammalian target of rapamycin (mTOR) signaling pathway (52). During the process of high glucose-induced cellular senescence, the expression and activity of SIRT1 protein are also significantly affected. Studies have found that high sugar leads to the downregulation of SIRT1 expression, thereby accelerating the senescence of endothelial cells. Additionally, compounds such as *Rutaecarpine*, an indoloquinazoline alkaloid from Evodia rutaecarpa, could attenuate high glucose-induced endothelial senescence by activating SIRT1, which deacetylates p53 and suppresses ROS generation (53). As has been evidenced previously, under conditions of high glucose, the level of ROS in cells significantly increases, leading to oxidative damage and cellular senescence (54). Additionally, high glucose induces cells to secrete inflammatory factors (such as IL-6 and TNF-α), exacerbating cellular senescence and dysfunction (55). The above studies provide important theoretical foundations for understanding the mechanisms of high glucose-induced cellular senescence.

## 3 The relationship between different types of rheumatic diseases and cellular senescence

### 3.1 RA and cellular senescence

#### 3.1.1 Overview of RA pathogenesis

RA is a chronic arthritic disease characterized by synovial proliferation, joint swelling and pain, and cartilage destruction, eventually leading to joint stiffness, deformity, and dysfunction, affecting about 12% of the global adult population (56, 57). Previous studies have suggested that oxidative stress, inflammation, hypercoagulable state, and apoptosis are closely related to RA pathogenesis (58–60). Growing evidence has suggested that there is a link between cellular senescence and RA pathogenesis (61).

#### 3.1.2 TS and genomic instability

TS is a hallmark of cellular senescence; although telomeres shorten physiologically with each cell division, this shortening rate can be increased by allostatic load and inflammatory insults, thus affecting the cellular senescence process (62). In a previous study of early and follow-up changes in leukocyte telomere length (rLTL) in RA patients, it has been found that rLTL shortening is associated with age, disease activity score (DET), and natural rLTL (63). In another multinational cohort study, researchers have demonstrated that TL shortening is closely associated with functional mutations of telomere-associated genes (TRGs) and senescence. Additionally, rTL is linked to baseline disease severity in RA-related interstitial lung disease (RA-ILD) (64). These observations are very similar to RA-ILD among U.S. Veterans; short TL is strongly associated with prevalent but not incident RA-ILD (65). These findings collectively support the view that TL shortening acts as a marker of accelerated RA senescence and underscore the importance of further research into TL variations.

#### 3.1.3 Bone cell senescence and joint destruction

In the process of bone resorption and metabolism, osteoclasts are mainly responsible for bone resorption and osteoblasts are responsible for bone formation (66). Osteoclasts are the major mediators of bone destruction, which are regarded as the main cell type of bone destruction in RA under pathological state (67). Bone destruction is the main feature of RA, which can result in osteoporosis. A previous study has shown that osteoblast replicative senescence in periarticular bones occurs more rapidly with senescence in RA than in OA patients, contributing to periarticular osteopenia in RA (68). This demonstrates that the replicative senescence of osteoblasts occurs more rapidly, exacerbating bone loss in RA. Cellular communication network factor 3 (CCN3), previously known as nephroblastoma overexpressed (NOV), is a secreted multifunctional protein involved in various cellular processes (69). A previous study has suggested that CCN3 is a new senescence marker of chondrocytes, and CCN3 overexpression in cartilage may partially promote chondrocyte senescence, leading to articular cartilage degeneration through the induction of p53 and p21 (70) (**Table 1**). Taiki et al. have manifested that CCN3 is highly expressed in RA joints and its level is associated with the severity of the disease (71). Further *in-vitro* assay demonstrated that stimulation with CCN3 resulted in the activation of the senescence pathway in fibroblast-like synoviocytes (FLS) and osteoclast differentiation in monocytes. These findings provide new potential targets for RA treatment, and future research can further explore the specific mechanisms of CCN3’s role in RA and its potential as a therapeutic target for preventing cellular senescence.

**Table 1:** Cellular senescence markers in rheumatic diseases.

Senescence indicators	Cells	Mechanism	Rheumatic diseases
IL-6	RA-FLS	Promoted inflammatory and immune	RA
MC1	RA-FLS	Activated ERK1/2 phosphorylation	RA
CCN3	RA-FLS	Suppressed inflammation and osteoclast numbers	RA
IL-6/IL-8	OA-CPCs	Increased production of intracellular ROS	OA
GPBAR1	OA-CPCs	Inhibited the expression of p21, p53, PAI-1, and the acetylation of SIRT1	OA
C12FDG	PBMCs	/	OA
TRB3	OA-CPCs	Induced chondrocyte autophagy and senescence in osteoarthritis cartilage	OA
ZMPSTE24	OA-CPCs	Affected chondrocyte metabolism	OA
LONP1	OA-CPCs	Through oxidative stress, metabolic changes and mitophagy, leading to MAPK pathway activation	OA
PKM2	OA-CPCs	Reduced senescent biomarker p16 INK4a expression	OA
MAPK12/FOS	OA-CPCs	Inhibited cell senescence and cartilage catabolism	OA
GDF15/MAPK14	OA-CPCs	Contributed to OA progression by inducing angiogenesis	OA
MiR-29b-5p	OA-CPCs	Suppressed the expression of MMP and P16INK4a/P21 through TET1	OA
MiR-140	OA-CPCs	Inhibited the expression of SA-βGal, p16INK4a, p21, p53, and γH2AX	OA
USP3	OA-CPCs	Upregulated SIRT3 to deacetylate FOXO3	OA
ARG2	OA-CPCs	Activated mTOR/S6K1 axis	OA
CYP1B1	MSC	Enhanced the stability of CYP1B1 mRNA and induced mitochondrial dysfunction	OA
MYL3	OA-CPCs	Inhibited clathrin-mediated endocytosis and activated Notch signaling	OA
SOX4	OA-FLS	ROS/TGF-β signal mediated accumulation of SOX4	OA
TGF-β/Alk5	OA-CPCs	SA-β-gal-positive cells and ROS production, swollen mitochondria and lysosome breakdown	OA
BHB	OA-CPCs	Facilitated the expression of PTEN by binding to hnRNP A1 and inhibiting the phosphorylation of Akt	OA
ASIC1a	OA-CPCs	Activated autophagy	OA
TK1	BMSCs	Enhanced IL-1β expression, apoptosis, cell cycle arrest, and senescent phenotypes	SLE
Leptin and NAP-2	MSCs	Activated PI3K/Akt signaling pathway	SLE
MiR-199a-5p	MSCs	Promoted splenic CD4+ T cell senescence through Sirt1/p53 pathway	SLE
Wnt/β-catenin	MSCs	Through the p53/p21 pathway	SLE
GRP 78	MSCs	Endoplasmic reticulum stress	SLE
JAK-STAT	MSCs	The cell volume and the number of SA-β-gal positive were increased	SLE
p16INK4a	T cell	Associated with higher fibrosis and CD8+ T cell infiltration	SLE
mTOR	MSCs	RAPA reversed the senescent phenotype and improved immunoregulation of MSCs from MRL/lpr mice and SLE patients	SLE
MAVS/IFNβ	MSCs	Decreased IFNβ, p53, and p16 protein levels	SLE
AOPPs	MSCs	Induced mitochondrial dysfunction resulted in elevated ROS	AS
WNT16	osteoprogenitor cells	Influenced in p21 protein and SASP mRNA expression	AS

#### 3.1.4 Synovial fibroblast senescence

The basic pathology of RA has been identified as a disorder of inflammation in the rheumatoid synovium, in which the predominant cell type is FLS (72, 73). The melanocortin 1 receptor (MC1R) is a member of the G-protein coupled receptor (GPCR) superfamily and belongs to the melanocortin (MC) subfamily of receptors (74). It has been shown that RA-FLS acquire a senescence phenotype after activating the MC1; selective activation of the GPCR MC1 may prevent the vicious cycle of reciprocal activation between FLS and macrophages within the inflamed joint environment, and then arise from the exploitation of senescence to target FLS that in RA fail to switch off (75). Moreover, through the GEO database, seven central genes (including IL-6) were identified, which are primarily associated with immune inflammation (76). Further experiments revealed that the inhibition of IL-6 significantly decreased the degree of FLS senescence. These findings demonstrated that RA-FLS senescence may promote RA development via inflammatory and immune mechanisms.

#### 3.1.5 Immune cell senescence

Many other senescent immune cells were found in RA lesions, contributing to the development or progression of RA. For example, it has been recently suggested that chronic inflammation-induced senescence impairs immunomodulatory properties of synovial fluid MSCs in RA, such as stemness, proliferation, cellular senescence, *in-vitro* differentiation, and *in-vivo* immunomodulatory properties (77). A previous study has proven that CD56(+) T cells with features of senescence in RA contribute to maladaptive immune responses, indicating that CD56(+) T cells are potential targets for therapy (78).

### 3.2 OA and cellular senescence

#### 3.2.1 Pathogenesis overview

**Figure 3:**
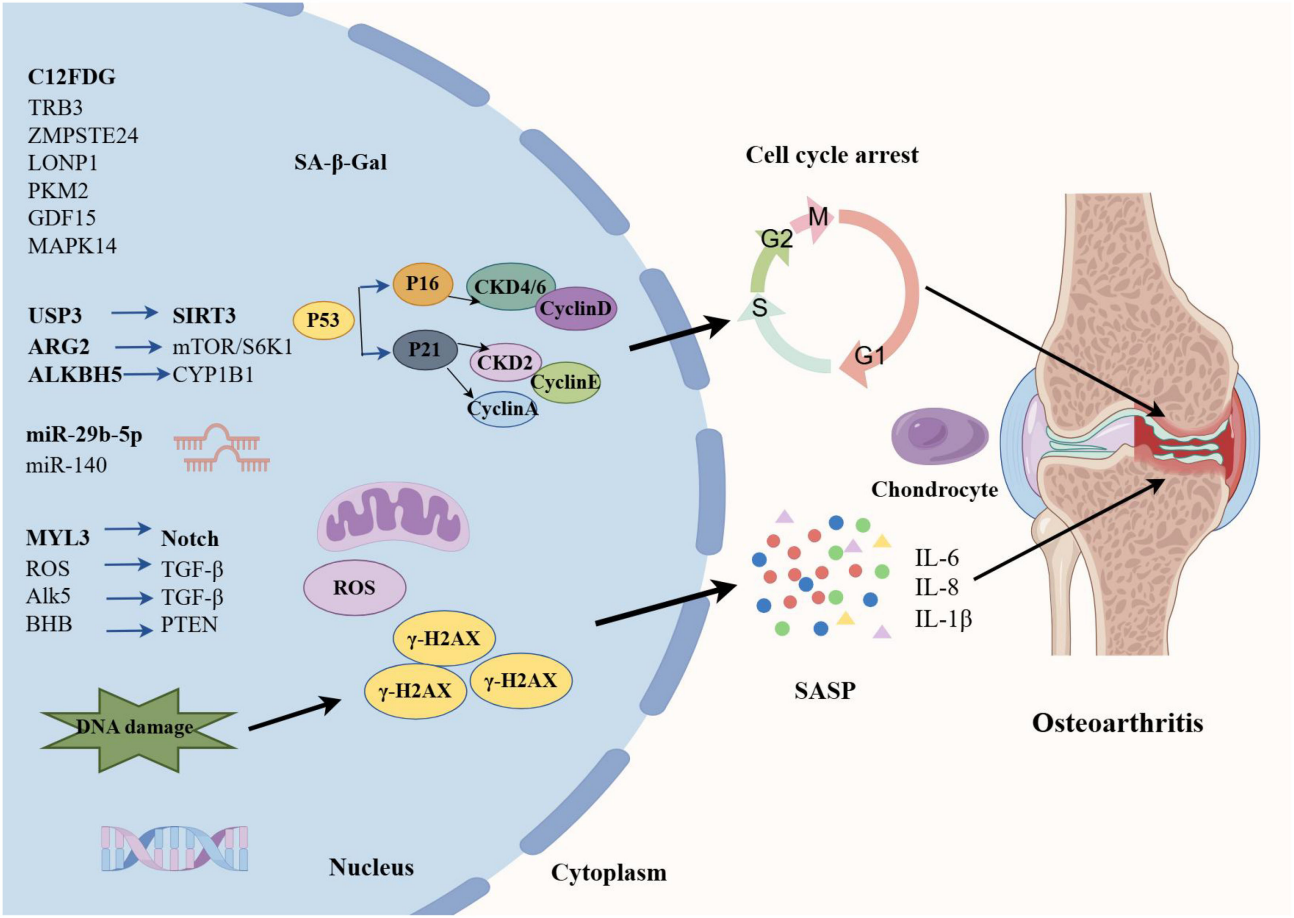
OA and cellular senescence by Figdraw.

OA is a chronic inflammatory disease closely related to cartilage degeneration characterized by joint pain, stiffness, joint swelling, and deformity, which significantly affect the quality of life of patients (79). OA has a complex pathogenesis involving multiple factors. Senescence is one of the primary causes of OA (80, 81) (**Figure 3**). As age advances, articular cartilage gradually degenerates, and the function of chondrocytes declines, leading to wear and destruction of cartilage. Additionally, obesity is also a significant risk factor for OA (82). Excessive body weight increases the burden on joints, especially the knee joint, thereby accelerating the degenerative process of cartilage. Joint injury is also a significant factor in the development of OA. Mechanical damage to joints can trigger local inflammatory responses, leading to further cartilage damage and degeneration (83).

#### 3.2.2 Senescence-associated secretory phenotype

The senescent cells increase the expression of cytokines, namely SASP (such as IL-β, IL-6, and TNF-α (84)), chemokines (such as MCP-1 and IL-8), cell adhesion molecules (such as E-selectin, ICAM-1, and VCAM-1), and MMP (such as MMP-2). A study about chondrogenic progenitor cells (CPCs) in OA has revealed that OA-CPCs exhibit elevated ROS levels along with a relatively high percentage of senescent cells, and senescence in OA-CPCs is mediated by the release of pro-inflammatory cytokines (such as IL-6 and IL-8) (85).

#### 3.2.3 Diagnostic biomarkers

With the advancement of cellular and molecular sciences, various molecules have been identified as cellular senescence-related molecules in OA, providing promising biomarkers for early diagnosis of OA and monitoring of OA progression and efficacy of treatments. For example, C12FDG+ PBMCs and T cells are elevated in OA patients, which may provide additional quantifiable clinical tools to assess healthy senescence and potential indicators for acute and chronic senescence-associated diseases in OA (86). Autophagy is a metabolic process that degrades damaged cells and proteins, and the defects in autophagy are closely associated with senescence (87). A previous study has evaluated the potential involvement of TRB3 in cartilage autophagy and cellular senescence in OA (88). They have concluded that TRB3 knockdown significantly rescues the reduced autophagy and elevated cell senescence in human chondrocytes. Therefore, interfering with TRB3 expression in cartilage may serve as a target for the prevention and treatment of OA cellular senescence. The deficiency of ZMPSTE24 could induce Hutchinson-Gilford progeria syndrome (HGPS), and it has been found that the expression of ZMPSTE24 is decreased in the articular cartilage of OA (89). Further transcriptome sequencing resulted in the deletion of ZMPSTE24 or affects chondrocyte metabolism, inhibits cell proliferation, and promotes cell senescence.

#### 3.2.4 Mitochondrial dysfunction

Mitochondrial dysfunction is strongly implicated in the cellular senescence mechanism, which is considered to be the culprit of cell senescence. Additionally, changes in mitochondrial function during senescence are considered to have a controlling role in cell fate (90, 91). A study has found that the expression of Lon protease 1 (LONP1), a mitochondrial protease, is decreased in human OA cartilage and senescent rat chondrocytes (92). LONP1 knockdown causes the imbalance of oxidative stress, metabolic changes, and mitophagy, leading to downstream MAPK pathway activation. These findings demonstrate that LONP1 is a central regulator of mitochondrial function in chondrocytes; downregulation of LONP1 with cellular senescence contributes to OA. Pyruvate kinase isoform M2 (PKM2) is one of the isoenzymes of pyruvate kinase and a key glycolytic enzyme. The role of PKM2 in senescence has also attracted significant attention. A recent study has indicated that the glycolytic enzyme PMK2 modulates chondrocyte senescence in OA but does not participate in the regulation of inflammation (93). MAPK12 and FOS have attracted widespread attention as potential senescence-related biomarkers. Through the analysis of OA-related datasets, researchers have identified significant expression changes in these two genes in the cartilage tissue of OA patients. Specifically, overexpression of MAPK12 and downregulation of FOS can promote cell proliferation and chondrocyte synthesis metabolism, and inhibit cell senescence and chondrocyte degradation metabolism, thereby alleviating the symptoms of OA (94). In addition, Weng et al. have shown that the GDF15/MAPK14 axis is a driver of senescent chondrocytes and can contribute to OA progression by inducing angiogenesis (95).

#### 3.2.5 Epigenetic regulation

microRNA (miR) expression involving epigenetic changes is one of the hallmarks of cellular senescence (96). Recently, it has been reported that senescence‐associated miR‐140 can effectively attenuate the progression of early-stage OA by retarding chondrocyte senescence (97). Specifically, pre-transfection with miR-140 could effectively inhibit the expression of cell senescence biomarkers (SA-β-Gal, p16INK4a, p21, p53, and γH2AX). Taken together, this study contributed new evidence for the involvement of miR-mediated epigenetic regulation of chondrocyte senescence in OA pathogenesis. Elsewhere, an experimental study has found that cellular senescence-related miRNA, miR-29b-5p, is markedly downregulated in OA cartilage, and their upregulation suppresses the expression of matrix metalloproteinases and senescence-associated genes (P16INK4a/P21) via TET1 (98).

Cellular senescence is characterized by various epigenetic changes, of which the mechanisms are mainly protein modification, such as acetylation, ubiquitination, and lactylation (99). m6A is a common RNA modification that plays an important role in regulating cell biological functions, and ALKBH5 is one of the key m6A demethylases (100). Recent research has demonstrated that downregulation of ALKBH5 expression facilitates MSC senescence by enhancing the stability of CYP1B1 mRNA and inducing mitochondrial dysfunction, revealing a novel mechanism of m6A in MSC senescence (101). Ubiquitination is a post-translational modification and a fundamental mechanism of proteostasis; ubiquitin-specific protease 3 (USP3) serves as a deubiquitinating enzyme to upregulate SIRT3 expression. A previous study has demonstrated that USP3 probably attenuates IL-1β-mediated chondrocyte senescence by deacetylating FOXO3 through SIRT3 (102). Protein lactylation, a novel PTM, is a new contributor to the epigenetic landscape and governs diverse physiological conditions (103). As has been evidenced previously, lactate accumulates in the pathological environment of OA, significantly upregulating the expression of arginase 2 (ARG2) and promoting the senescence of OA-FLS by activating the mTOR/S6K1 signaling pathway (104). This senescence is characterized by features such as G1/S phase arrest, increased ROS, and β-galactosidase production. These results suggested that the ARG2-mTOR/S6K1 axis plays an important role in lactate-induced OA-FLS senescence and that regulating this signaling pathway can provide new targets and therapeutic strategies for treating OA and other senescence-related diseases.

#### 3.2.6 Main signaling pathways

Various signaling pathways participate in the cellular senescence process. For example, it has been shown that the Notch signaling pathway plays a crucial role in cell fate determination (105). In the Notch signaling process, the endocytosis mediated by clathrin is an important regulatory step. A previous study has shown that the absence of MYL3 leads to enhanced iridium-mediated endocytosis, which further promotes Notch internalization and activation of the Notch signaling (106), thereby accelerating the cellular senescence process of OA chondrocytes. Moreover, SOX4 acts as a key transcription factor in the senescence process of OA- FLS. It has been shown that the activation of SOX4 promotes cellular senescence and the formation of SASP in OA-FLS, a process regulated by the ROS/TGF-β signaling (107). Therefore, in-depth research on the regulatory mechanisms and functions of SOX4 in cellular senescence is expected to provide new insights and targets for the treatment of diseases (such as OA). Another study has determined that the TGF-β/Alk5 signaling prevents OA initiation by regulating the senescence of articular cartilage stem cells (108). Similarly, it has been found that β-hydroxybutyrate (BHB) exhibits anti-senescence effects in various cellular senescence-related diseases. A study has shown that BHB could alleviate the senescence of OA-Chos and slow down the progression of OA (109). GO enrichment analysis revealed significant changes in cell cycle genes, with PTEN being the most significantly differentially expressed gene. BHB promotes PTEN expression by binding to hnRNP A1 and inhibiting Akt phosphorylation, thereby alleviating the senescence of chondrocytes.

#### 3.2.7 Autophagy-senescence crosstalk

Autophagy plays a crucial role in cellular senescence, especially under oxidative stress conditions (110, 111). Oxidative stress can influence cellular autophagy activities through multiple pathways, thereby affecting the senescence process of cells (112). A previous study has shown that acid-sensitive ion channel 1a (ASIC1a) participates in acid-induced chondrocyte autophagy, and also inhibits senescence-related markers, suggesting that ASIC1a may be involved in acid-induced OA articular chondrocyte senescence by activating autophagy (113). A study has directly compared the different effects of oxidative and inflammatory stresses on the senescence of rat chondrocytes, and they have found that intra-articular injections of H2O2 cause no change in senescent markers; however, IL/TNF injections increase the proportion of p16- and SASP factor-positive OA chondrocytes (114).

#### 3.2.8 Comparison of immunosenescence between RA and OA

RA and OA are two distinct joint disorders that differ significantly in etiology, pathogenesis, and immunological characteristics, especially in the context of immunosenescence. RA is an autoimmune disease marked by systemic immune activation, involving aberrant lymphocyte responses, persistent inflammation, and features of accelerated immunosenescence (such as the expansion of senescent T cells, TS, and excessive production of SASP factors). In contrast, OA is primarily a degenerative joint disease characterized by localized cartilage degradation and comparatively mild immune involvement, whereas low-grade inflammation is mainly mediated by macrophages and selected cytokines. Despite these differences, both RA and OA share some overlapping features, including infiltration of immune cells (such as macrophages and T cells) into affected joints, and engagement of common inflammatory pathways. However, the magnitude and nature of immune responses differ significantly, with RA involving more pronounced lymphocyte activation and systemic immune dysfunction. It’s critical to understand these distinctions, as OA is often used as a comparative model in RA studies. Clarifying the shared and unique aspects of immunosenescence in these diseases provides deeper insights into their respective pathophysiologies and may aid in identifying disease-specific therapeutic targets.

### 3.3 SLE and cellular senescence

#### 3.3.1 Disease overview and pathogenic mechanisms

SLE is a systemic autoimmune disease with various pathogenic factors and involvement of multiple organs, as well as diverse clinical manifestations and a relapsing and remitting course, among which the kidney is one of the most commonly involved organs (115). SLE predominantly affects young women, and its pathogenesis involves various factors, including genetic predispositions, environmental triggers (such as ultraviolet light), and immune system abnormalities (116). SLE patients have various autoantibodies in their bodies, which not only target nuclear antigens but may also target other cellular components. These autoantibodies bind to antigens to form immune complexes, which deposit in tissues and trigger inflammatory responses and tissue damage (117). The complement system also plays a crucial role in the pathogenesis of SLE. Deficiencies in the early components of the complement system can lead to improper clearance of immune complexes and apoptotic cell debris, thereby promoting the onset of SLE. Additionally, excessive activation of the complement system may also result in organ damage (118). Moreover, cellular senescence plays a crucial role in the pathogenesis of SLE (119). During the natural course of SLE, the ongoing process of chronic immune activation and pro-inflammatory phenotypes can lead to immunosenescence (120). Studies have shown that premature senescence of immune cells occurs in SLE patients, which may be associated with mitochondrial dysfunction, abnormal immune metabolism, and telomere/telomerase imbalance. For example, in a cohort study by Sahena et al., it has been concluded that TL is shortened in SLE patients, which is significantly associated with Ro antibody positivity (121). Therefore, in-depth research on the mechanisms of cellular senescence in SLE is not only helpful for understanding its pathogenesis but may also provide clues for developing new therapeutic strategies.

#### 3.3.2 T cell senescence and dysfunction

The activation of T cells plays a crucial role in the pathogenesis of SLE. Studies have shown that there are abnormalities in T cell signaling in SLE patients, which can lead to hyperactivation of T cells and disruption of self-tolerance, thereby promoting disease progression (122). Additionally, the number of Th17 cells increases while that of regulatory T cells (Tregs) decreases in SLE patients, and this imbalance between Th17/Tregs is also considered an important factor in the pathogenesis of SLE (123). The CD57 antigen and killer cell lectin-like receptor G1 (KLRG1) are markers commonly defined by the terminally differentiated T cells, which are also senescence markers. As has been evidenced previously, SLE patients show notably higher senescence T cell markers compared to the controls, and the increase of T cell senescence, especially in the CD8 compartment, is correlated with the disease activity and severity of SLE (124). The other two studies have respectively mentioned that T cellular senescence is related to early arteriosclerosis and angiogenesis in SLE. A study has shown that T cell cellular senescence markers (such as CD4+CD57+, CD8+CD57+, CD4+CD28null, and CD8+CD28null) are also significantly higher in SLE patients, showing a significant positive correlation with the level of atherosclerotic markers (cIMT, FMD, and soluble adhesion molecules) (125). These results suggested that T cell cellular senescence may be a significant factor in early atherosclerosis in SLE patients. Another study has revealed the existence of an aberrant CD28null-angiogenic T cell population in SLE patients, which is characterized by a senescent and cytotoxic phenotype, and is associated with anti-dsDNA titer and increased production of pro-inflammatory mediators (126). Wang et al. have shown that the frequency of CD4+CD57+ senescent T cells is notably elevated and is positively correlated with disease activity, indicating that CD4+CD57+ senescent CD4+ T cells play a dominant role in the pathogenesis of SLE and hold promise for developing a potential treatment for ameliorating lupus phenotypes (127). Induction of senescence in T cells in SLE is a potential therapeutic approach. SIRT1 is a NAD-dependent deacetylase that protects against cell senescence by deacetylating p53. It has been shown that miR-199a-5p can promote the senescence of CD4+ T cells in the spleen by regulating the SIRT1/p53 pathway, thereby alleviating lupus symptoms in MRL/lpr mice. The core of this mechanism lies in the crucial role of the SIRT1/p53 pathway in cellular senescence and immune regulation (128). These findings provide new insights and potential targets for the treatment of SLE.

#### 3.3.3 MSC senescence

MSCs have shown significant potential in the treatment of SLE and other autoimmune diseases, especially in cellular senescence terms (129). Studies have demonstrated that MSCs can modulate the immune system through various mechanisms, thereby suppressing the autoimmune response in SLE patients. Firstly, MSCs possess potent immune regulatory capabilities, as they can reduce the production of pro-inflammatory cytokines by inhibiting the proliferation of T cells and B cells, thus alleviating inflammatory responses. Additionally, MSCs can induce the generation of regulatory Tregs and restore immune balance, thereby mitigating the pathological progression of SLE. In clinical application, the transplantation of MSCs has been proven to improve renal function and other organ damage in SLE patients (130). Chen et al. have identified that TK1 expression is remarkably elevated in SLE bone marrow-derived MSCs (BM-MSCs), and silencing TK1 could alleviate inflammation, growth arrest, and senescence in BM-MSCs of SLE, highlighting TK1’s considerable promise as a therapeutic target against SLE (131). The Wnt/β-catenin signaling plays an important role in stem cell senescence. A study has also demonstrated that the Wnt/β-catenin signaling may play a critical role in the senescence of SLE BM-MSCs through the p53/p21 pathway (132). Therefore, elucidating the mechanisms of the Wnt/β-catenin signaling involved in the senescence of SLE BM-MSCs will help improve the transplantation efficacy of BM-MSCs in SLE. Furthermore, another study has underscored the pivotal role of the JAK-STAT signaling in regulating the senescence of BM-MSCs in SLE (133). They have found that AG490, the inhibitor of JAK2, and knockdown of STAT3 in BM-MSCs, could significantly reverse the senescence. Interestingly, the PI3K/AKT signaling also emerges as a key factor in the implementation of cellular senescence. It has been discovered that leptin and neutrophil-activating peptide 2 (NAP-2) act synergistically to promote MSC senescence through enhancement of the PI3K/Akt signaling pathway in SLE patients. Additionally, the mTOR pathways and inhibition of the mTOR signaling pathways of rapamycin, are closely related to SLE cellular senescence (134). Gu et al. have demonstrated that RAPA reverses the senescent phenotype and improves immunoregulation of MSCs in MRL/lpr mice and SLE patients through inhibition of the mTOR signaling pathway (135). Additionally, the role of the IFNβ signaling in systemic SLE is increasingly recognized, especially in cellular senescence. It has been evidenced that IFNβ signaling forms a positive feedback loop with mitochondrial antiviral signaling (MAVS), a mechanism that plays a crucial role in the senescence phenotype of SLE BM-MSCs (136). These findings suggest that the IFNβ signaling pathway may become a new target for SLE treatment. The activation of endoplasmic reticulum stress (ERS) is involved in the growth arrest in the G1 phase of the cell cycle. Research has revealed that ERS-mediated senescence is a critical determinant of BM-MSCs in SLE patients (137).

#### 3.3.4 Organ-specific senescence manifestations

SLE can lead to damage to multiple organs and systems, such as lupus nephritis and lupus encephalopathy. Neuropsychiatric manifestations (NP) are common in SLE patients, which is closely related to cellular senescence (138). A previous study has considered that SLE patients develop an accelerated immunosenescence which contributes to cognitive dysfunction, especially in attention, recall, and visuospatial domains (136). Lupus nephritis is characterized by inflammation in the kidney, proteinuria, and progressive kidney damage. Hence, there is a need to better understand the mechanisms underlying disease progression. The increase in p16INK4a-positive cells has been shown to be closely related to renal fibrosis and CD8+ T cell infiltration, suggesting that cellular senescence may play a crucial role in the pathogenesis of LN (139).

### 3.4 AS and cellular senescence

AS is a chronic inflammatory disease primarily characterized by sacroiliac joint and spinal erosion. Patients may experience joint pain, stiffness, and limited range of motion, and in severe cases, spinal deformity and joint fusion (140). Moreover, AS is strongly associated with human leukocyte antigen B27, which predominantly affects young and middle-aged males (141). Currently, there is no specific curative medication for AS, and biological agents are the primary drugs for AS treatment. However, these biologics are often expensive and may have numerous side effects. The introduction of multiple biologics targeting TNF-α and IL-17 marks a significant advancement in the management of AS, providing relief for many patients who have not responded to conventional therapies (such as NSAIDs and physiotherapy) (142, 143). Despite their effectiveness, biologics drugs still have some limitations. Hence, it’s necessary to explore alternative therapies.

It has been pointed out that oxidative stress exists in the serum environment of AS patients, which can lead to the senescence of MSCs. Oxidative stress promotes the senescence of MSCs by inducing mitochondrial dysfunction and excessive ROS production (144). This process not only exacerbates the decline in cell function but may also trigger inflammatory responses, thereby contributing to the pathological progression of AS. WNT16 is critical for bone homeostasis, which is pivotal for the differentiation and bone formation of osteoblasts. It has been revealed that WNT16 is highly expressed in AS, and WNT16 treatment facilitates cellular senescence in AS-osteoprogenitor cells during osteoblast differentiation accompanied by suppression of bone formation (145). In addition, premature senescence of T-cell subsets in axial spondyloarthritis has been demonstrated in a prospective study (146). Specifically, this study demonstrates an age-inappropriate shrinkage of thymic output, and an inappropriate shortening of telomeres in young patients with aSpA. All the above studies evidence that cellular senescence is closely related to the development of AS.

### 3.5 SS and cellular senescence

SS is a systemic autoimmune disease characterized by vasculopathy, inflammation, and fibrosis of the skin and internal organs, often resulting in severe disability and high mortality (147). The pathogenesis of SS is complex, and many mechanisms have not yet been clarified. Although it is inappropriate to consider SS as a disease of senescence, the possible role of cellular senescence in SS pathogenesis should be considered as an important factor (148). There is strong evidence that inflammation and oxidative stress may be involved in this process (149, 150).

Accumulating studies have indicated that the alterations in TL play a role in the development of various autoimmune diseases. There are very few studies on TL in SS. Artlett et al. have found that the average loss of telomeric DNA in SS patients and family members is 3 kb; however, this loss is not related to age, but is caused by chromosomal instability (151). In another study, Tarhan et al. have demonstrated a very low telomerase activity in SS compared with that in other connective tissue diseases. These results indicated that the important role of cellular senescence in SS has been emerging (152). A cross-sectional study supports the significant association between cellular senescence and SS; they have found that endothelial-to-mesenchymal transition (EndMT) and fibroblast senescence are more abundant in skin biopsies of SSc patients. Specifically, cellular senescence may accelerate the fibrosis process by promoting the activation of fibroblasts and excessive deposition of extracellular matrix (ECM) (153). These findings indicate that both senescence and EndMT are involved in the pathway leading to skin fibrosis in SS, and may be valuable biomarkers and/or possible targets for novel therapeutic interventions.

ILD is the most common pulmonary manifestation in SS patients and is the most frequent cause of SSc disease-related death (154). A recent gene expression meta-analysis has revealed cellular senescence signatures in SS-ILD (155). They have suggested that markers (GDF15, COMP, and CDKN2A) and pathways (p53) of senescence are significantly increased in SS-ILD; moreover, TL in SSc-ILD is comparable to idiopathic pulmonary fibrosis. This study represents an important step forward toward a better understanding of the cellular senescence association of SS, particularly with lung involvement. In SS patients, MSCs not only maintain their pluripotency and proliferative capacity but also normally induce regulatory Tregs with functional phenotypes. Tregs play crucial roles in maintaining immune tolerance and suppressing excessive immune responses, which is of great significance for cell-based therapeutic strategies. As previously revealed, SS-MSCs show an increase in senescence biomarkers; moreover, the increased activation of the IL-6 pathway observed in our cells may represent an adaptive mechanism to senescence, while preserving Tregs functions, including immunosuppression (156).

## 4 Treatment strategy for rheumatic diseases from the perspective of cellular senescence

In recent years, therapeutic strategies targeting cellular senescence have become a focal point in anti-senescence research, including drugs that selectively clear senescent cells (known as senolytics) and drugs that inhibit SASP and other senescent markers (known as senomorphics). In preclinical studies, senolytics have shown significant potential. For example, research has found that senolytics can induce apoptosis in senescent cells by inhibiting specific proteasome pathways, thereby reducing SASP secretion and improving tissue function (157). Additionally, researchers have developed senolytic prodrugs that can be specifically activated *in vivo* through interaction with senescence biomarkers, thereby reducing drug side effects and enhancing therapeutic efficacy (158).

Phoenixin-20 (PNX-20) is a peptide targeting G-protein-coupled receptor 173 (GPR173) with promising anti-inflammatory properties. A recent study has found that PNX-20 protects the TNF-α-induced cell senescence in RA-FLSs by downregulating STAT6, suggesting that PNX-20 may be a promising agent for the treatment of RA (159) (**Table 2**). Apremilast is a phosphodiesterase 4 (PDE4) inhibitor that has therapeutic potential in various inflammatory diseases. SIRT1 is an important deacetylase that plays a crucial role in cellular senescence and inflammatory responses (160). In this study, Apremilast has been found to prevent IL-17-induced cellular senescence in ATDC5 chondrocytes by modulating the SIRT1 pathway, which provides a new idea and potential therapeutic strategy for the treatment of cartilage degeneration-related diseases (161). Navitoclax (ABT263) is a well-known anti-senescence drug that can alleviate symptoms of OA by selectively eliminating senescent cells (162). Using Navitoclax to clear these senescent cells can effectively reduce the levels of inflammatory mediators, decrease the activity of matrix-degrading enzymes, and protect cartilage tissue. Therefore, as a potential therapeutic approach, Navitoclax can alleviate symptoms of OA and promote the regeneration and repair of cartilage by targeting senescent cells.

**Table 2:** Anti-senescence drugs for rheumatic diseases.

Drugs	Targets	Mechanism
Phoenixin-20	STAT6	Decreased telomerase activity, decreased the expression level of p21 and p53
Apremilast	SIRT1/IL-17	Reduced the expression of IL-1β, MCP-1, and ROS, inhibited the expression of p21 and PAI-1
Navitoclax	Casepase3/P21	Improved inflammatory microenvironment and promoted cartilage phenotype maintenance
Jianpi qingre tongluo prescription	STAG1/TP53/P21	Regulated SASP factors, and restored ECM balance
Huangqin Qingre Chubi Capsules	p53/p21	Inhibited inflammatory response and ECM degradation
Quzhi Tang	GPNMB/Nrf2/NF-κB	Reduced intracellular oxidative stress and restored impaired mitochondrial function
Forsythoside A	PKC/Nrf2	Attenuated the pathological signs of knee OA
Panaxatriol	UFL1	Delayed OA progression and reduced cartilage repair fibrosis

Traditional Chinese medicine (TCM) has a multi-pathway, multi-target, and multi-level mechanism of action in treating senescence, which makes it highly regarded in anti-senescence research (163). Importantly, TCM can effectively delay the senescence process by modulating some signaling pathways (such as AMPK/mTOR, FOXO, and SIRT1). These signaling pathways play crucial roles in cellular energy metabolism, oxidative stress response, and autophagy, thereby improving the overall senescence state of the organism. Huangqin Qingre Chubi Capsules (HQC), a Chinese patent medicine prepared by the First Affiliated Hospital of Anhui University of Traditional Chinese Medicine, strengthens the spleen and resolves dampness, and clears heat and unblocks collaterals (164). The latest research has suggested that HQC modulates SASP factors by regulating the STAG1/TP53/P21 signal transduction axis and decelerating cartilage senescence and degradation in OA patients (165). A previous study has also demonstrated that HQC delays chondrocyte senescence in OA rats, and inhibits inflammatory response and ECM degradation by inhibiting the p53/p21 pathway in experimental animals (166). Quzhi Tang (QZT) is a compound formula composed of six TCM herbs, which has achieved good clinical outcomes in treating KOA. A previous study has shown that QZT can regulate the GPNMB/Nrf2/NF-κB signaling pathway, repair mitochondrial damage, and improve the senescence process of chondrocytes (167). The *in-vivo* experiments have demonstrated that interfering with GPNMB expression can reduce mitochondrial dysfunction and cellular senescence while increasing Nrf2 expression and decreasing NF-κB expression levels. Treatment with QZT can increase Nrf2 expression, decrease NF-κB expression, repair mitochondrial damage, and improve the degree of chondrocyte senescence. Fruitauin A (FTA) is a phenylethanolide extracted from the fruit of Forsythia suspensa, exhibiting significant anti-inflammatory and antioxidant properties. Furthermore, the *in-vitro* experiments have revealed that FTA improves mitochondrial function, enhances mitochondrial autophagy, inhibits the activation of NLRP3 inflammatory bodies, and suppresses chondrocyte senescence. Additionally, this study has found that FTA directly binds to Nrf2 and enhances its phosphorylation through PKC (168). Ubiquitin-fold modifier 1-specific E3 ligase 1 (UFL1) has been identified as a novel target of panaxatriol for anti-senescence effects. A previous study has shown that panaxatriol inhibits chondrocyte senescence through the UFL1/forkhead box O1 (FOXO1)/P21 and UFL1/NF-κB/SASPs signaling pathways (169).

Despite the promising performance of these therapeutic strategies in preclinical studies, there are still some challenges in clinical application. Researchers are exploring how to translate these small molecule senolytics and other anti-senescence interventions into clinical practice to play a preventive or therapeutic role in rheumatic diseases.

## 5 Conclusions and prospects

Overall, this review explored the link between cellular senescence and rheumatic diseases. Despite significant efforts to develop effective treatments for rheumatic diseases, a definitive solution remains elusive. The review highlighted the importance of understanding cellular senescence, senescence-associated secretomes, and their interactions with rheumatic diseases. Targeting cellular senescence has emerged as a significant strategy in the treatment of rheumatic diseases. By reducing the burden of senescent cells, senolytics can mitigate the inflammatory environment and improve tissue function, offering a novel therapeutic avenue for managing rheumatic diseases. However, there are still some limitations, which are elaborated as follows.

First, the lack of specific markers for cellular senescence is a major obstacle. Although some studies have identified some potential markers (such as p16INK4a and β-galactosidase), they are not always specific and may behave differently in different cell types and tissues. Furthermore, the heterogeneity of senescent cells increases the complexity of identification, as different cell types may exhibit distinct characteristics and functions during the senescence process.

Second, the senescent cells may negatively impact the microenvironment of surrounding tissues by secreting pro-inflammatory cytokines and other factors, thereby exacerbating disease progression. Therefore, developing technologies and strategies that can effectively identify and eliminate senescent cells is crucial for slowing the progression of cellular senescence-related diseases. In short, despite some progress in the study of cellular senescence, further exploration and development of specific markers and recognition techniques are needed to better understand the role of senescent cells in physiological and pathological processes and provide new ideas for the treatment of rheumatic diseases.

Third, the challenges in applying nanotechnology to cellular senescence are multifaceted. One major challenge is the need for precise targeting of senescent cells without affecting normal cells. This requires the development of highly specific nanocarriers that can recognize and bind to markers unique to senescent cells. Additionally, the safety and biocompatibility of nanomaterials must be thoroughly evaluated to prevent adverse effects. Additionally, nanoparticle-induced inflammatory responses (such as generation of ROS and activation of the NF-κB signaling pathway) need to be carefully managed to avoid exacerbating cellular damage. Despite these challenges, the potential benefits of nanotechnology in combating cellular senescence are significant. By improving the delivery and efficacy of anti-senescence therapies, nanotechnology could play a crucial role in preventing rheumatic diseases.

An increasing number of studies have provided promising indications of a causal link between cellular senescence and the onset and progression of rheumatic diseases. However, it is crucial to expand research efforts in both basic science and clinical applications to unlock the full potential of targeting senescent cells.
